# The Electrically Silent Kv6.4 Subunit Confers Hyperpolarized Gating Charge Movement in Kv2.1/Kv6.4 Heterotetrameric Channels

**DOI:** 10.1371/journal.pone.0037143

**Published:** 2012-05-17

**Authors:** Elke Bocksteins, Alain J. Labro, Dirk J. Snyders, Durga P. Mohapatra

**Affiliations:** 1 Laboratory for Molecular Biophysics, Physiology and Pharmacology, Department of Biomedical Sciences, University of Antwerp, CDE, Universiteitsplein 1, Antwerpen, Belgium; 2 Department of Pharmacology, The University of Iowa Roy J. and Lucile A. Carver College of Medicine, Iowa City, Iowa, United States of America; Indiana University School of Medicine, United States of America

## Abstract

The voltage-gated K^+^ (Kv) channel subunit Kv6.4 does not form functional homotetrameric channels but co-assembles with Kv2.1 to form functional Kv2.1/Kv6.4 heterotetrameric channels. Compared to Kv2.1 homotetramers, Kv6.4 exerts a ∼40 mV hyperpolarizing shift in the voltage-dependence of Kv2.1/Kv6.4 channel inactivation, without a significant effect on activation gating. However, the underlying mechanism of this Kv6.4-induced modulation of Kv2.1 channel inactivation, and whether the Kv6.4 subunit participates in the voltage-dependent gating of heterotetrameric channels is not well understood. Here we report distinct gating charge movement of Kv2.1/Kv6.4 heterotetrameric channels, compared to Kv2.1 homotetramers, as revealed by gating current recordings from mammalian cells expressing these channels. The gating charge movement of Kv2.1/Kv6.4 heterotetrameric channels displayed an extra component around the physiological K^+^ equilibrium potential, characterized by a second sigmoidal relationship of the voltage-dependence of gating charge movement. This distinct gating charge displacement reflects movement of the Kv6.4 voltage-sensing domain and has a voltage-dependency that matches the hyperpolarizing shift in Kv2.1/Kv6.4 channel inactivation. These results provide a mechanistic basis for the modulation of Kv2.1 channel inactivation gating kinetics by silent Kv6.4 subunits.

## Introduction

Voltage-gated K^+^ (Kv) channels are K^+^ selective membrane spanning multimeric channel proteins with ion-conducting pores that actively open, close or inactivate in response to changes in the membrane potential. They are critical determinants of cellular excitability since they contribute to the shape, duration and frequency of action potentials and can also contribute to the regulation of resting membrane potential [1]. Kv channels exist as tetramers of α-subunits each containing six transmembrane segments (S1–S6). The S5–S6 segments of each α-subunit assemble to form the central K^+^ selective pore while the S1–S4 segments form the voltage sensing domains (VSD) that surround this central pore domain [2]. Within the VSDs, the positively charged S4 segments form the main voltage-sensing components that move outward upon membrane depolarization, which further translates into structural rearrangements to open the channel gate in order to allow electrodiffusion of K^+^ ions across the membrane [1], [3], [4], [5], [6], [7]. The movement of the S4 charges across the transmembrane electrical field results in a transient charge (Q) displacement that can be recorded as a gating current (*I*_Q_), which has been considered as the direct measure of the voltage-dependence of channel gating [3]. Opening of the channel gate itself occurs in a concerted step when all four VSDs have moved to their activated state [7], [8], [9]. Although the voltage-dependency of Kv channel gating is directly controlled by the VSDs of the pore forming α-subunits, a number of modulatory and/or auxiliary subunits can modify channel gating properties, which ultimately influence the cellular excitability *in vivo* [10], [11].

The electrically silent Kv channel α-subunit Kv6.4 is not capable of forming functional homotetrameric channels; however, it can heterotetramerize with Kv2.1 α-subunits to form functional Kv2.1/Kv6.4 channel complexes, presumably in a 3∶1 stoichiometry [12], [13]. Kv6.4 exerts several changes in the biophysical properties of Kv2.1 in Kv2.1/Kv6.4 channel complexes: a decrease in the current density [14] and a hyperpolarizing shift in the voltage-dependence of inactivation by ∼40 mV, but without any significant effects on voltage-dependence of channel activation [15]. Here we show the modulating effects of Kv6.4 on Kv2.1 gating properties by analyzing the voltage-dependence of VSD movements in Kv2.1 and Kv2.1/Kv6.4 channels from *I*_Q_ recordings. Our results suggest that Kv6.4 subunits display an intrinsic voltage-dependency with an operational VSD in heterotetrameric Kv2.1/Kv6.4 channels, by virtue of which it specifically influences the voltage-dependent inactivation properties of Kv2.1.

## Results

### Heterotetrameric Kv2.1/Kv6.4 channels exhibit advanced gating charge movement

**Figure 1:**
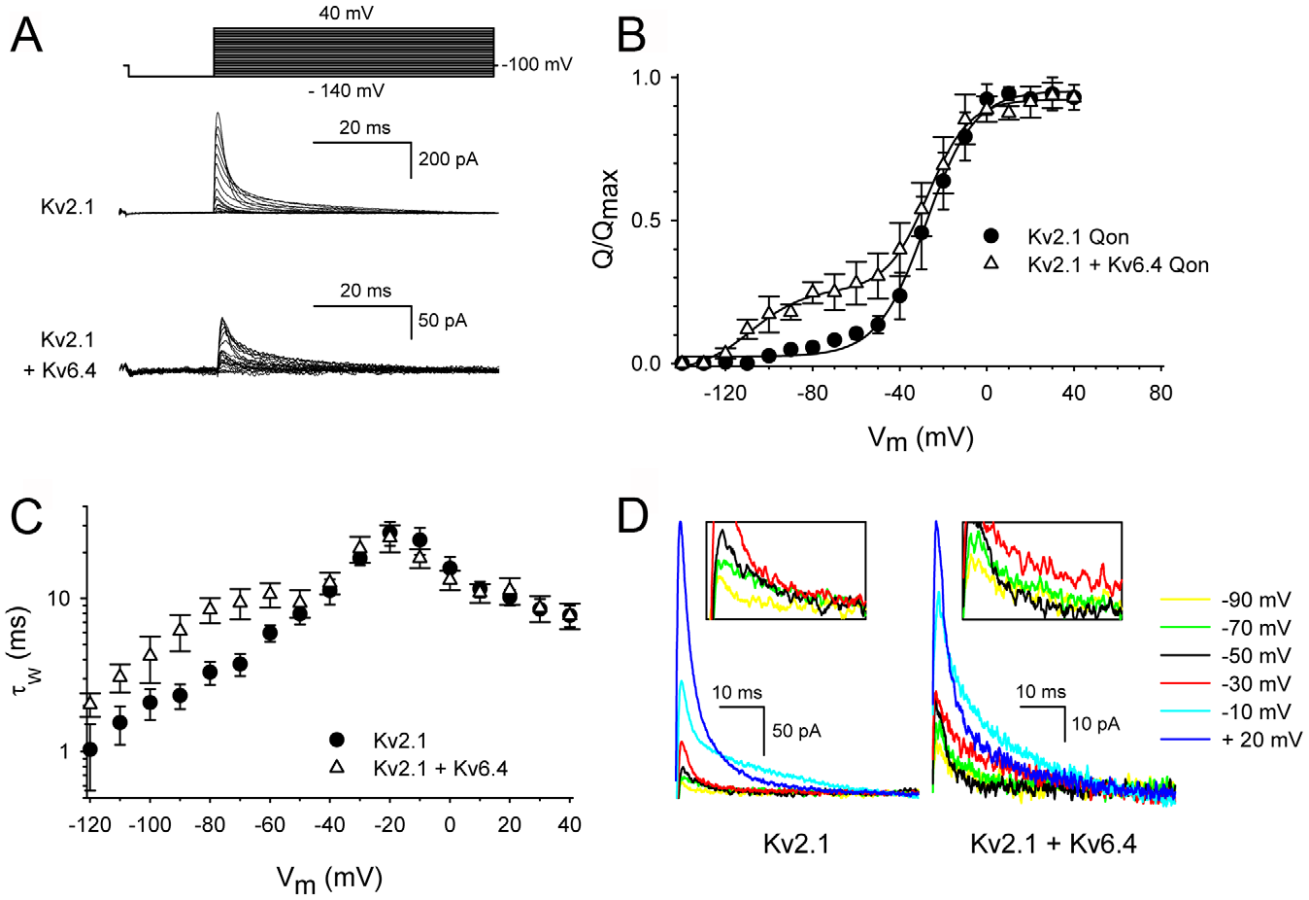
Gating current properties of Kv2.1 and Kv2.1/Kv6.4 channels. (A) Representative ON-gating current recordings from Kv2.1 and Kv2.1/Kv6.4 channels. (B) Boltzmann fits of Q–V curves for Kv2.1 alone (circle) and upon co-expression with Kv6.4 (triangle). (C) Voltage-dependence of the weighted time constants of *I*_Q_ of Kv2.1 alone (circle) and upon co-expression with Kv6.4 (triangle). *I*_Q_ kinetics was obtained by fitting the *I*_Q_ decay from the current recordings shown in panel c. (C) Scaled up view of *I*_Q_ currents at different voltages for Kv2.1 alone (left) and upon co-expression with Kv6.4 (right). The inset highlight the crossing at −50 mV observed for Kv2.1/Kv6.4 channels.

We recorded *I*_Q_ currents (Figure 1A) from cells expressing Kv2.1 homotetramers or Kv2.1/Kv6.4 heterotetramers and determined the voltage-dependence of the ON-gating charge (Q) movement, which showed a sigmoidal relationship that could be fitted with a single Boltzmann function (Q–V curve; Figure 1B). The voltage for half-maximal displacement of ON-gating charge (Q_1/2_) of Kv2.1 homotetrameric channels was −26.5±4.7 mV (Figure 1B, Table 1), which is consistent with previous observations [16], [17]. This corresponds to an apparent charge movement of 3.8 electronic charges as determined from the Q–V slope (as described in materials and methods, Figure 1B, Table 1). Furthermore, the bell-shaped voltage-dependence of the time constants of *I*_Q_ decay reached a maximum around −20 mV, which corresponds to the Q_1/2_ potential (Figure 1C, Table 1). Compared to the ionic conductance-voltage (G–V) relationship of Kv2.1 [10], [13], [14], [16], [18], [19], the Kv2.1 Q–V curve was displaced by ∼30–40 mV towards more hyperpolarized potentials, which is expected for a channel that possesses multiple close states before opening [17], [20].

**Table 1:** Ionic and gating current properties of Kv2.1 alone and upon co-expression with Kv6.4. Values are given as mean ± s.e.m.

	1^st^ component		2^nd^ component		n
	Q_1/2_ (mV)	k	Q_1/2_ (mV)	k	
Q–V curve					
Kv2.1	n.a.	n.a.	−26.5±4.7	4.6±0.5	8
Kv2.1+Kv6.4	−93.2±6.0	9.4±2.4	−21.8±4.2	5.8±1.8	7
τ_w_ (ms)	at −70 mV		at −20 mV		
Kv2.1	3.0±0.5		16.5±2.2		8
Kv2.1+Kv6.4	5.8±0.9		14.5±2.8		7

For comparison the Kv2.1 parameters are shown under “2^nd^ component” when two components are obtained with the Kv2.1/Kv6.4 expression.

Interestingly, the Q–V relationship of Kv2.1/Kv6.4 heterotetrameric channels showed a specific and different voltage-dependence of Q_ON_ movement, with an additional component in the Q–V curve representing ∼20% of the total charge moving below −40 mV (Figure 1B). Approximation with a double Boltzmann function yielded a first component with a Q_1/2_ potential of −93.2±6.0 mV and an apparent charge movement of 1.8 electronic charges, and a second component with a Q_1/2_ potential of −21.8±4.2 mV and an apparent charge movement of 3.0 electronic charges (Figure 1B, Table 1). While the second component displayed a voltage-dependency similar to that of Kv2.1 homotetrameric channels, the first component was displaced approximately −70 mV in hyperpolarizing direction, and therefore, most likely represents the gating charge movement of Kv6.4 subunits. To assure that this first component could originate from Kv6.4 subunits, we ensured that we could detect K^+^ ion conduction of heterotetrameric Kv2.1/Kv6.4 channels under the same conditions used to record the Kv2.1/Kv6.4 gating currents (Figure S1, Table S1). However, under these conditions (i.e. higher cDNA concentrations) the ionic currents could only be controlled with minimal voltage error when 70 mM tetraethyl ammonium chloride (TEA-Cl) was added to the extracellular buffer. In addition to the second gating component observed in the Q–V curve of heterotetrameric Kv2.1/Kv6.4 channels, the voltage-dependence of the time constants of Kv2.1/Kv6.4 *I*_Q_ decay displayed a double bell-shaped curve with two maxima, one around −70 mV and the other one around −20 mV (Figure 1C, Table 1). This strengthens the presence of two gating components whereby the kinetics associated with the first component appeared to be slightly faster than those of the second one, the latter one matching the kinetics of Kv2.1 homotetramers.

Due to this bell-shaped relationship between the kinetics of *I*_Q_ decay and voltage, the gating charge movement in Kv2.1 homotetramers slowed down between −120 and −20 mV and accelerated again with stronger depolarizations. This resulted in a conspicuous crossover of the current traces in Kv2.1 homotetramers with depolarizing potentials above −10 mV (Figure 1D, Table 1). This is in agreement with previous reports on the gating current behavior of other Kv channels [19]. However, in case of the Kv2.1/Kv6.4 heterotetramers, where the time constants displayed a double bell-shaped relation, two such phases are to be expected. Indeed, there was a crossing of the current traces in the voltage range between −70 mV and −30 mV. Above −30 mV the current decay slowed down and crossing disappeared. Finally, with depolarizations above −10 mV the crossing of the currents reappeared (Figure 1D, Table 1). Based on prior observations from our and several other groups, no silent Kv subunit homotetramers have been detected in the cell plasma membrane [10], due to which we did not perform *I*_Q_ current recordings from cells expressing Kv6.4 alone.

### Kv6.4 effects on Kv2.1 gating can be predicted with a simplified gating model

**Figure 2:**
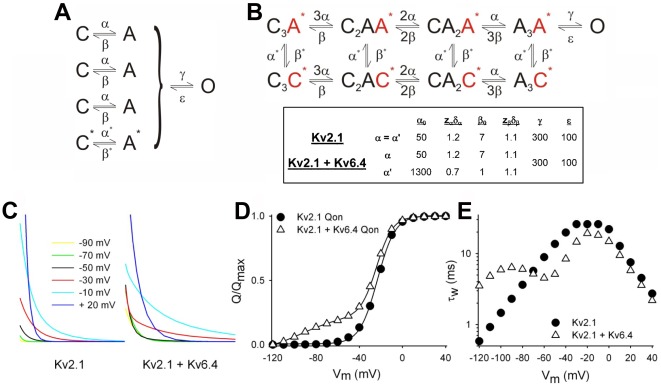
Simulation of Kv2.1 and Kv2.1/Kv6.4 gating currents. (A) Scheme depicting the simplified model in which the activation of each subunit was modeled with a single closed (C) to activated (A) transition with an exponential voltage-dependence of the microscopic rate constants with parameters as detailed in panel B. Once all four subunits are in the A state, transition to the open (O) state occur in a final (voltage independent) step (B) Markov model used for the simulation of Kv2.1 and Kv2.1/Kv6.4 gating currents. In case of Kv2.1 homotetramers, C* = C, A* = A, and α* = α, and β* = β. For the heterotetramer C* and A* represent the Kv6.4 subunit in the Kv2.1/Kv6.4 in 3∶1 stoichiometry with distinct parameters for α* and β*. The values used to simulate the gating currents with this model are given in the box below. For further details on these models see the materials and methods section. (C) Simulated *I*_Q_ gating currents at different potentials for the Kv2.1 homotetramer (left) and Kv2.1/Kv6.4 heterotetramer (right) using the model shown in panel a. Note the crossing at −50 mV for the Kv2.1/Kv6.4 heterotetrameric channel. (D) Q–V curves for Kv2.1 (circle) and Kv2.1/Kv6.4 (triangle) obtained by integrating the simulated *I*_Q_ shown in panel B and fitted with Boltzmann function. (E) Voltage-dependence of the weighted time constants of *I*_Q_ of Kv2.1 (circle) and Kv2.1/Kv6.4 (triangle) channels obtained by fitting the *I*_Q_ decay from the simulated *I*_Q_ shown in panel C. Note the negative component in both the Kv2.1+Kv6.4 Q–V curve and *I*_Q_ kinetics which correspond well with the experimental data in figure 1.

The data above showed that compared to Kv2.1 homotetramers, Kv2.1/Kv6.4 heterotetrameric channels have an extra gating component at more negative potentials in both the Q–V curve and the time constants of *I*_Q_ decay. To test whether a single Kv6.4 subunit with a more negative voltage-dependency can generate the observed *I*_Q_ behavior of Kv2.1/Kv6.4 heterotetrameric channel, we used computational modeling with a Markov state model depicted. For convenience we used a simplified gating model (Figure 2A) with a single transition between closed (C) and activated (A) state for each subunit, followed by the concerted step into the open (O) state (after all four subunits have reached the A-state). The equivalent Markov state-model (Figure 2B) was built such that it could simulate both the heterotetrameric Kv2.1/Kv6.4 channel configuration with a 3∶1 stoichiometry, as well as the homotetrameric Kv2.1 channel. To represent the heterotetrameric stoichiometry the closed and activated state of the Kv6.4 subunit are indicated with asterisks (C* and A*, respectively). To simulate the homotetrameric Kv2.1 channel the rate constants of the C* to A* transition were equal to those of the C to A transition. Figures 2C–E illustrates that this minimal model could reproduce the key features of experimentally obtained *I*_Q_ behavior of both the Kv2.1 homotetramers and the Kv2.1/Kv6.4 heterotetramers. In the latter case there was indeed charge movement linked to the VSD movement of the Kv6.4 subunit (Figures 2D–E) at much more negative potentials, together with the effect on the *I*_Q_ decay kinetics. Analysis of the state occupancies in the model linked this charge movement effectively to the VSD movement of the Kv6.4 subunit.

## Discussion

Silent Kv channel α-subunits fail to form homotetrameric Kv channels, but form functional Kv channels upon heterotetramerization with functional Kv2 channel α-subunits [10], [11]. This poses a critical question of whether the VSDs of silent Kv channel α-subunits are functional in heterotetrameric complexes, i.e. whether they contribute in any way to the voltage-dependency of channel gating. Our results show that there was an additional component in the VSD movement at hyperpolarized potentials upon co-expression of Kv6.4 with Kv2.1 (Figure 1B, Table 1). Nevertheless, the effect of Kv6.4 on the channel's activation gating are limited, as deduced from similarity of the G–V relationship of the channel, compared to that of Kv2.1 [13], [14]. This can be explained on the basis that gate opening occurs in a final concerted step when all four VSDs have been activated [7], [8], [9]. Therefore, the intrinsic voltage-dependency of the Kv6.4 subunit is only reflected at the level of charge movement and appears to be shifted towards more negative potentials compared to Kv2.1. A Kv2.1 chimera with the S4 segment substituted by its Kv6.4 counterpart resulted in channels with a voltage-dependency that likewise was shifted towards more hyperpolarized potentials [15], supporting that the negative component in our gating current recordings reflects properties of the S4 segment of Kv6.4 subunits.

Remarkably, the voltage-dependency of the Kv6.4 subunit appears to correspond with the strong (−40 mV) hyperpolarizing shift in the voltage-dependence of steady-state inactivation of Kv2.1/Kv6.4 heterotetrameric channels, as compared to Kv2.1 homotetramers. If the 3∶1 stoichiometry proposed for Kv2.1/Kv9.3 [12] also applies to Kv2.1/Kv6.4 heterotetramers, these results suggest that the VSD movement of a single Kv6.4 subunit is sufficient to initiate channel inactivation. For the *Shaker* channel it has been suggested that early transitions between closed states are accompanied with structural rearrangements within the bottom section of S6 [21], which in case of Kv6.4 might thus be sufficient to trigger channel inactivation.

Previously, it has been determined that a single Kv2.1 channel possesses a gating charge of 12.5 electronic charges which is essentially equal to that from *Shaker* and Kv1.1 channels [22]. Due to limitations in channel expression (due to Kv6.4-induced drastic reduction in Kv2.1 current density) we had to co-express Kv2.1 and Kv6.4 in a 1∶1 ratio instead of the preferred 1∶3 ratio that is needed to ensure the presence of only Kv2.1/Kv6.4 heterotetramers [14], instead of a mixed population of Kv2.1 homotetramers and Kv2.1/Kv6.4 heterotetramers (Figure S1). Consequently, we cannot exclude the presence of homotetrameric Kv2.1 channels within the total channel population (Figure S1), and therefore, we were not able to determine the gating charge contribution of a single Kv6.4 subunit. To obtain an indication for the amount of gating charge we measured the slope of the Q–V curve that reflects the apparent amount of charge moved per subunit [7]. The slope of the Q–V curve obtained for the homotetrameric Kv2.1 channels yielded an apparent charge movement of 3.8 electronic charges (e^−^), which in a four-fold symmetric channel would result in a total apparent charge movement of 15 e^−^. This is somewhat higher than the established gating charge of 12.5 e^−^ but indicates that this slope-method is a reasonable indicator of the total charge. Interestingly, while comparing the slopes of both components in the Q–V curve of the Kv2.1/Kv6.4 heterotetramer, we found that the apparent gating charge of the most negative component (reflecting the Kv6.4 subunit) was less than that of the Kv2.1 subunit; 1.8 e^−^ versus 3.0 e^−^, apparent gating charge, respectively. In *Shaker* channels it has been established that only the four most extracellularly located S4 Arginine residues (R1–R4) cross (at least a part of) the transmembrane field and contribute to the channel's gating charge [23], [24]. A sequence alignment between Kv2.1 and Kv6.4 shows that R4 is missing in Kv6.4 (replaced by a tyrosine), which might explain the observed difference in gating charge. Interestingly, the slope of the Kv6.4-induced component in the voltage-dependence of channel inactivation is also shallower than this of Kv2.1 [10], [14] (Figure S1), which is in agreement with the observed difference in the apparent amount of gating charge and the slope of the Q–V curve.

**Figure 3:**
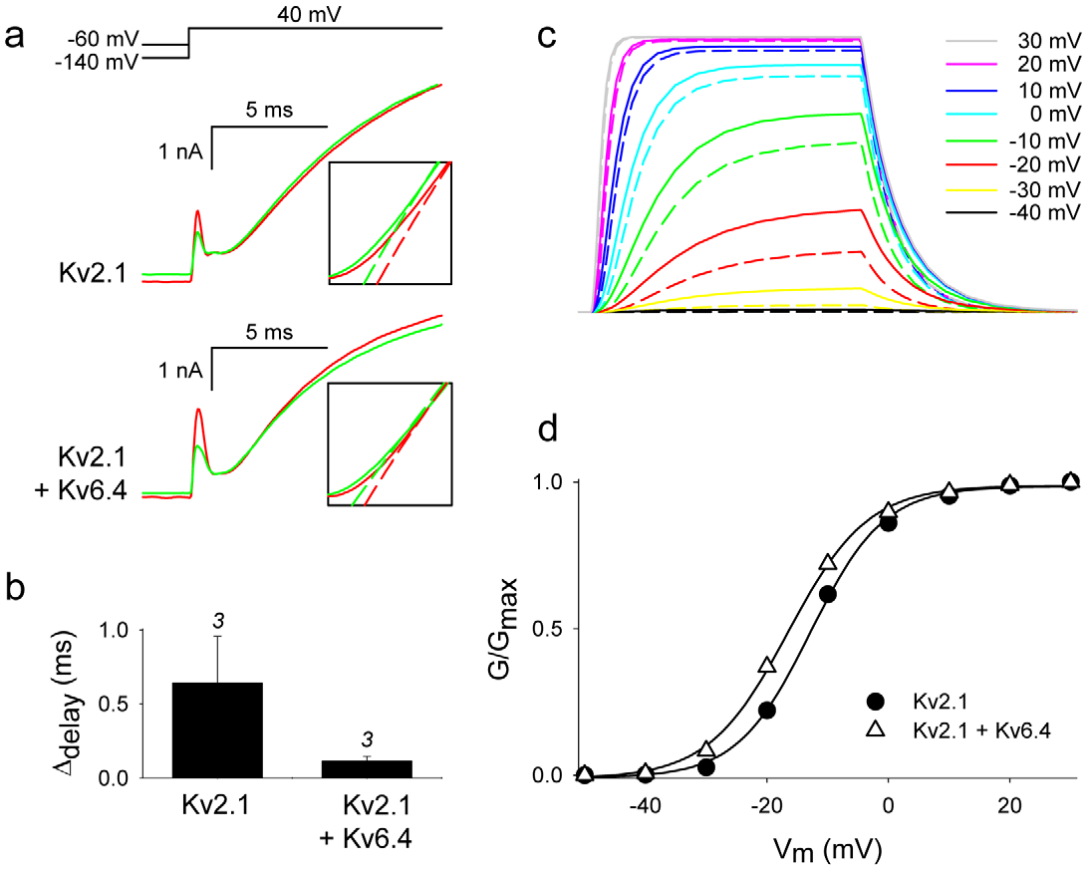
Cole-Moore shift in homotetrameric Kv2.1 and heterotetrameric Kv2.1/Kv6.4 ionic currents. (A) Representative ionic currents of Kv2.1 homotetramers (middle) and Kv2.1/Kv6.4 heterotetramers (bottom) elicited by the pulse protocol shown at the top. The insets highlight the delay in current activation upon a prepulse potential of −140 mV (red trace) or −60 mV (green trace). The dashed lines represent the single exponential fit of the raw current traces. Note the obvious increase in the delay (i.e. Cole-Moore shift) of current activation in Kv2.1 homotetramers between a prepulse potential of −60 mV and −140 mV, respectively. For the Kv2.1/Kv6.4 heterotetrameric configuration this delay (i.e. Cole-Moore shift) is markedly less pronounced. (B) Bar chart representations of the time difference in current activation delay using a holding of −140 mV as compared to a −60 mV holding. This time difference (Cole-Moore shift) is substantially smaller in the heteromeric Kv2.1/Kv6.4 channel. The figure above every bar indicates the number of cells analyzed. (C) Simulated ionic currents at different potentials for the Kv2.1 homotetramer (dashed lines) and Kv2.1/Kv6.4 heterotetramer (full lines) using the model shown in figure 2A. Note the acceleration in activation of the Kv2.1/Kv6.4 heterotetramers compared to the Kv2.1 homotetramers. (D) Voltage-dependence of activation of Kv2.1 alone (circles) and upon co-expression with Kv6.4 (triangles) obtained by plotting the simulated peak ionic currents in panel C against the respective membrane voltage.

It has been established that for channel opening the individual channel subunits traverse through several different closed states before reaching the activated conformation. When all four subunits have reached the activated state, channel opening proceeds in a concerted cooperative way [3], [8]. The existence of multiple voltage-dependent closed states results in a Cole-Moore shift in the time course of activation after strong hyperpolarizing potentials [25]. During such strong hyperpolarizations the subunits populate deeper closed states. This causes the delay between membrane depolarization and channel gate opening to be prolonged as a consequence of passing through more closed states. In homotetrameric Kv2.1 channels such a prolonged delay in channel opening (i.e. the Cole-Moore shift) was obvious when comparing activation after a prepulse potential of −140 mV and −60 mV, respectively (Figure 3A). In the heterotetrameric channels, the Kv6.4 subunit is to a large extent in the activated configuration at −80 mV due to the negatively shifted voltage-dependency. Therefore this Cole-Moore shift should be reduced at the same holding potentials [26]. Although a prepulse potential of −60 mV already induced channel inactivation, the Cole-Moore shift was indeed less pronounced for the Kv2.1/Kv6.4 homotetramers compared to the Kv2.1 homotetramers (Figure 3A–B). Furthermore, although our simplified gating model is not suited to study Cole-Moore shifts in detail because it contains only a single closed state, it predicts that heterotetrameric Kv2.1/Kv6.4 channels open earlier and display a ∼4 mV negative shift in the G–V curve compared to Kv2.1 homotetramers (Figure 3C–D). The experimental data concur with the model and show indeed a 3 mV hyperpolarizing shift in the voltage-dependency of channel opening that is often reported as not significant and neglected (Figure 3D). These data - i.e. a reduced Cole-Moore shift and a small shift in the G–V curve of heterotetrameric Kv2.1/Kv6.4 channels - further support the notion that the Kv6.4 subunit displays gating charge movement at more hyperpolarized potentials compared to Kv2.1 subunits.

In the context of a mixed population of channels, it can be argued that the observed negative component in the *I*_Q_ recordings is a reflection of an altered voltage-dependence of Kv2.1/Kv6.4 heterotetrameric channels as a whole and not that of the silent Kv6.4 subunit, specifically. However, this would imply that only the early transitions in VSD movement would be altered in the heterotetramer, without affecting the final concerted step that leads to channel gate opening (i.e. a phenomenon that is similar to the reported ILT mutations in Shaker [27]). Although we cannot fully exclude this, imposing this within a 3∶1 channel stoichiometry is not evident. Furthermore, based on the voltage-dependence of channel inactivation the overall distribution of homotetrameric and heterotetrameric channels appeared to be roughly equal but the contribution of the negative component in the total charge being moved is only 20%. This strengthens our hypothesis that this negative gating component indeed reports on the intrinsic voltage-dependency of the silent Kv6.4 subunit that manifests itself at the level of ionic currents by a −40 mV shift in the voltage-dependence of inactivation. In conclusion, we provide a molecular explanation for the mechanism by which the silent Kv6.4 subunit modulates the voltage-dependent gating properties of the Kv2.1 channel.

## Materials and Methods

### Molecular biology, and transfection of mammalian cells

The cDNAs of recombinant rat Kv2.1 and human Kv6.4 were inserted into the mammalian expression vector pRBG4 [18], [19] following standard molecular biology methods. HEK293A cells (Invitrogen, San Diego, CA) were cultured in Dulbecco's Modified Eagle's medium supplemented with 10% fetal bovine serum, 2 mM glutaMAX and 100 units/ml of penicillin-streptomycin (Invitrogen). In contrast to parent HEK293 cells the HEK293A cells adhere strongly to the culture dishes and coverslips, which is why these cells were used for the electrophysiological experiments in this study. Cells were transiently transfected with plasmids containing Kv2.1 (for homotetrameric channels) or Kv2.1 and Kv6.4 (for heterotetrameric channels), along with the peGFP-c1 plasmid as a selection marker for transfection, using the Lipofectamine2000 reagent according to manufacturer's instructions (Invitrogen). Forty-eight hours post transfection cells were used in electrophysiological experiments.

### Electrophysiology

Gating current (*I*_Q_) recordings were obtained from HEK293A cells transfected with above-mentioned channel constructs, under whole-cell configuration using an Axopatch-200B amplifier connected to a Digidata 1440A data acquisition system, and controlled with the pClamp10 software (Molecular Devices, Sunnyvale, CA). Current recordings were sampled at 1 to 10 kHz and filtered at 1 kHz with a low-pass Bessel filter. Patch pipettes were pulled using PC-10 puller (Narishige International USA, East Meadow, NY), from borosilicate glass tubes (World Precision Instruments, Sarasota, FL) and then heat polished at the tip to give a resistance of 3–6 MΩ, when filled with the intracellular solution. The extracellular solution for *I*_Q_ recordings contained (in mM) 140 TEA-Cl, 5 KCl, 2 CaCl_2_, 1 MgCl_2_, 10 HEPES, 10 Glucose and adjusted to pH 7.3 with NaOH, and the cells were superfused continuously. The intracellular solution contained (in mM) 2 NaCl, 140 NMDG, 1 CaCl_2_, 1 MgCl_2_, 5 EGTA, 3 Mg-ATP, 0.3 Na-GTP and 10 HEPES with the pH adjusted to 7.3 using NaOH. All the chemicals used in extracellular and intracellular buffers were purchased from Sigma-Aldrich, St. Louis, MO. *I*_Q_ recordings were performed after the depletion of K^+^ with repeated depolarizing pulses of +40 mV for 500 ms from a resting potential of −100 mV for 25–50 times. For *I*_Q_ recordings cells were depolarized for 60 ms from −140 to +40 mV with +10 mV increments after a 20 ms prepulse to −140 mV from a holding potential of −100 mV. Background leak and capacitive currents were subtracted using a P/8 protocol. For the details about cell transfections, recording solutions and voltage protocols utilized in ionic current recordings see supplementary text S1.

### Data Analysis

For *I*_Q_ recordings, the area under the *I*_Q-ON_ for the entire 60 ms pulse duration at each voltage were determined as the total gating charge, normalized to the maximal gating charge, and plotted against the respective membrane voltage, as described earlier [17]. The voltage-dependence of gating charge movement was fitted with a Boltzmann equation according to *y = 1/[1+exp(-(V-Q_1/2_)/k)]*, in which *V* represents the applied voltage, *Q_1/2_* the voltage at which 50% of the charge is moved, and *k* the slope factor. The amount of electronic charges that has been moved has been determined with the equation z = k_B_*T/slope*e, in which *z* represents the amount of electronic charge, *k_B_* the Boltzmann constant, *T* the absolute temperature, *slope* the slope of the fitted Q–V curve and *e* the electronic charge [7]. Kinetics of charge movement were fitted with a single or double exponential function and represented as weighted time constants. Results are presented as mean ± s.e.m for each data point. For the details about analysis of ionic current recordings and calculation of voltage-dependence of channel activation and steady-state inactivation see supplementary text S1.

### Computational modeling

Gating current simulations were obtained with a multi-state Markov model solved with the Q-matrix approach in Matlab™. The main purpose was to test whether the inclusion of 1 subunit with a voltage-dependence of activation that is shifted in hyperpolarized direction would reproduce the key experimental findings. Therefore we used a simplified model (Figure 2A) in which the activation of each subunit was modeled with a single closed (C) to activated (A) transition with an exponential voltage-dependence of the microscopic rate constants with parameters as detailed in figure 2B.

The Markov model (Figure 2B) is the integrated state representation in which for the fourth subunits the rate constants can be assigned to be WT (i.e. C* = C, A* = A, α* = α and β* = β) for Kv2.1 homotetramers or to be distinct for the heterotetramer with a 3∶1 stoichiometry. The voltage-dependence of α and β was modeled with a single voltage-dependent transition represented by a log-linear voltage-dependence *α = α_o_exp(e_o_z_α_δ_α_E/kT)* and *β = β_o_exp(-e_o_z_β_δ_β_E/kT)* in which *α_o_* and *β_o_* reflect the transition rate constants at 0 mV, *k* is the Boltzmann constant, *T* is the absolute temperature, *E* is the voltage, *e_o_* is the elementary charge and *z_α_δ_α_* or z*_β_δ_β_* are the apparent charge movements for these transitions [3]. The numerical values are provided in figure 2A. Simulations started with all states at their equilibrium-state at the holding potential.

## Supporting Information

Text S1 **Additional electrophysiological methods.**
(PDF)

Figure S1 Ionic current properties of Kv2.1 and Kv2.1/Kv6.4 channels. (A) Representative whole cell ionic current recordings of Kv2.1 alone (middle row) and upon co-expression with Kv6.4 in a 1∶1 transfection ratio (lower row) elicited by the pulse protocol given in the top row. (B) Boltzmann fits of the voltage-dependence of activation (filled) and steady-state inactivation (unfilled) of Kv2.1 alone (circles) and upon co-expression with Kv6.4 (triangles). For comparison, the Boltzmann fit of the voltage-dependence of steady-state inactivation of Kv2.1 upon co-expression with Kv6.4 in a 1∶3 transfection ratio (square) is also shown. Peak ionic currents at each depolarizing pulse (for activation) or at the test pulse after each conditioning pulse (for steady-state inactivation), were taken for the analysis of voltage-dependence of channel activation/inactivation, plotted against the respective membrane voltage, and fitted with a Boltzmann function (solid line) as described earlier [14], [16], [18], [19].
(PDF)

Table S1 Ionic current properties of Kv2.1 alone and upon co-expression with Kv6.4. Values are given as mean ± s.e.m. For comparison the Kv2.1 parameters are shown under “2^nd^ component” when two components are obtained with the Kv2.1/Kv6.4 expression.
(PDF)
