# Supplementary material for: The Electrically Silent Kv6.4 Subunit Confers Hyperpolarized Gating Charge Movement in Kv2.1/Kv6.4 Heterotetrameric Channels
Source: PLoS One. 2012 May 17;7(5):e37143. doi: 10.1371/journal.pone.0037143 (PMC3355112; doi:10.1371/journal.pone.0037143)
Supplement: Text S1 — Additional electrophysiological methods. (PDF) [file pone.0037143.s001.pdf]

SUPPLEMENTARY TEXT S1:

Additional electrophysiological methods: Ionic currents were obtained from HEK293A cells transfected with 1 µg Kv2.1 alone (for homotetrameric channels) or upon co-expression with Kv6.4 (in a 1:1 or 1:3 ratio, for heterotetrameric channels), along with the peGFP-c1 plasmid as a transfection marker, using the Lipofectamine2000 reagent according to manufacturer's instructions (Invitrogen, San Diego, CA). Current recordings were performed in the whole-cell configuration with an Axopatch-200B amplifier (Axon instruments) connected to a Digidata 1400 data acquisition system (Axon instruments). The current recordings were sampled at 1 to 10 kHz and filtered at 1 kHz with a low-pass Bessel filter with. The pClamp10 software (Molecular Devices) was used to control command voltages and data storage. Patch pipettes were pulled from borosilicate glass tubes (World Precision Instruments, Sarasota, FL) and then heat polished at the tip to give a resistance of 3-6 MΩ, when filled with the intracellular solution. Ionic current recordings were obtained with an extracellular solution containing (in mM) 70 NaCl, 70 tetraethyl ammonium (TEA)-Cl, 5 KCl, 2 $CaCl_2$, 1 $MgCl_2$, 10 HEPES, 10 Glucose and adjusted to pH 7.3 with NaOH, and an intracellular solution containing (in mM) 2 NaCl, 140 KCl, 1 $CaCl_2$, 1 $MgCl_2$, 5 EGTA, 3 MgATP, 0.3 NaGTP and 10 HEPES with the pH adjusted to 7.3 using KOH. After obtaining whole-cell configuration, capacitance and series resistance were compensated (80%). Recordings with voltage errors originating from the series resistance exceeding 5 mV were excluded from analysis. Transfection of 1 µg Kv2.1 alone or in combination with Kv6.4 in HEK293A cells led to robust expression of Kv channels that is necessary for I_Q current recordings; however, the K^+ ionic currents under such circumstances were at a magnitude of >50 nA at positive potentials. Therefore, TEA-Cl was used in the extracellular buffer to reduce the K^+ conductance to a level that could be recorded with a minimal voltage error. Under such circumstances the G-V relationship exhibited ~10 mV hyperpolarizing shift, as compared to prior reports [14,16,18,19], which could presumably be due to the presence of TEA-Cl in the extracellular solution, or due to relatively dephosphorylated

Kv2.1 channels in HEK293A cells (not shown), as compared to Kv2.1 expressed in HEK293 cells [18,19,28].

For steady-state activation parameters, currents were recorded with serial depolarization of cells for 500 ms from -80 to +80 mV with +10 mV increments, from a holding potential of -70 mV. At the end of each depolarizing pulse, a deactivating pulse at -45 mV for 250 ms, followed by repolarization to holding potential was applied, in order to attain complete deactivation of channels. For steady-state inactivation parameters, currents were recorded with serial conditioning pulse for 5 s from -110 to +40 mV with +10 mV increments, from a holding potential of -70 mV, followed by a test pulse at +60 mV for 250 ms. Interpulse intervals in activation and inactivation parameter recording protocols were 15 s and 30 s, respectively. The voltage-dependence of channel activation and inactivation, as well as the voltage-dependence of gating charge movement were fitted with a Boltzmann equation according to $y = 1/[1+exp(-(V-V_{1/2})/k)]$, in which V represents the applied voltage, $V_{1/2}$ the voltage at which 50% of the channels are activated or inactivated, and k the slope factor.

SUPPLEMENTARY REFERENCE:

28. Mohapatra DP, Park KS, Trimmer JS (2007) Dynamic regulation of the voltage-gated Kv2.1 potassium channel by multisite phosphorylation. Biochem Soc Trans 35: 1064-1068.
