# Supplementary figures and images for: The Electrically Silent Kv6.4 Subunit Confers Hyperpolarized Gating Charge Movement in Kv2.1/Kv6.4 Heterotetrameric Channels
Source: PLoS One. 2012 May 17;7(5):e37143. doi: 10.1371/journal.pone.0037143 (PMC3355112; doi:10.1371/journal.pone.0037143)

Figure S1

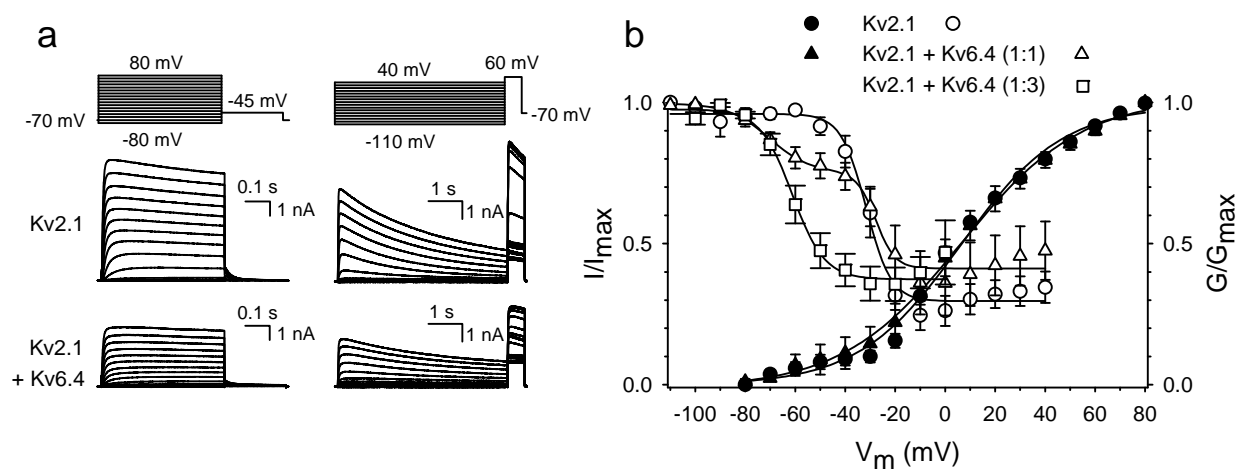

Supplement: Figure S1 — Ionic current properties of Kv2.1 and Kv2.1/Kv6.4 channels. (A) Representative whole cell ionic current recordings of Kv2.1 alone (middle row) and upon co-expression with Kv6.4 in a 1∶1 transfection ratio (lower row) elicited by the pulse protocol given in the top row. (B) Boltzmann fits of the voltage-dependence of activation (filled) and steady-state inactivation (unfilled) of Kv2.1 alone (circles) and upon co-expression with Kv6.4 (triangles). For comparison, the Boltzmann fit of the voltage-dependence of steady-state inactivation of Kv2.1 upon co-expression with Kv6.4 in a 1∶3 transfection ratio (square) is also shown. Peak ionic currents at each depolarizing pulse (for activation) or at the test pulse after each conditioning pulse (for steady-state inactivation), were taken for the analysis of voltage-dependence of channel activation/inactivation, plotted against the respective membrane voltage, and fitted with a Boltzmann function (solid line) as described earlier [14], [16], [18], [19]. (PDF) [file pone.0037143.s002.pdf]
