# Supplementary material for: The Electrically Silent Kv6.4 Subunit Confers Hyperpolarized Gating Charge Movement in Kv2.1/Kv6.4 Heterotetrameric Channels
Source: PLoS One. 2012 May 17;7(5):e37143. doi: 10.1371/journal.pone.0037143 (PMC3355112; doi:10.1371/journal.pone.0037143)
Supplement: Table S1 — Ionic current properties of Kv2.1 alone and upon co-expression with Kv6.4. Values are given as mean ± s.e.m. For comparison the Kv2.1 parameters are shown under “2nd component” when two components are obtained with the Kv2.1/Kv6.4 expression. (PDF) [file pone.0037143.s003.pdf]

Supplementary Table 1

	1st component		2nd component		n
	$V_{1/2}$ (mV)	k	$V_{1/2}$ (mV)	k	
GV curve					
Kv2.1	4.9 ± 3.0	19.8 ± 1.4	n.a	n.a	7
Kv2.1 + Kv6.4	2.5 ± 6.8	18.3 ± 0.9	n.a	n.a	6
Inactivation					
Kv2.1	n.a	n.a	-32.9 ± 3.0	4.1 ± 0.4	6
Kv2.1 + Kv6.4	-72.0 ± 2.6	8.0 ± 1.1	-25.1 ± 2.8	3.7 ± 0.1	5
